# Nocturnal Smartphone Use Affects Sleep Quality and Cognitive and Physical Performance in Tunisian School-Age Children

**DOI:** 10.3390/ejihpe14040055

**Published:** 2024-03-28

**Authors:** Rihab Abid, Achraf Ammar, Rami Maaloul, Mariem Boudaya, Nizar Souissi, Omar Hammouda

**Affiliations:** 1 Research Unit: Physical Activity, Sport, and Health, UR18JS01, National Observatory of Sport, Tunis 1003, Tunisia; n_souissi@yahoo.fr; 2 Department of Training and Movement Science, Institute of Sport Science, Johannes Gutenberg University Mainz, 55122 Mainz, Germany; 3 Interdisciplinary Laboratory in Neurosciences, Physiology and Psychology: Physical Activity, Health and Learning (LINP2), UFR STAPS, Faculty of Sport Sciences, UPL, Paris Nanterre University, 92000 Nanterre, France; omar.hammouda@parisnanterre.fr; 4 Research Laboratory, Molecular Bases of Human Pathology, LR19ES13, Faculty of Medicine, University of Sfax, Sfax 3029, Tunisia; rami.maaloulll@gmail.com; 5 Biochemistry Laboratory, CHU Hedi Chaker, University of Sfax, Sfax 3000, Tunisia; mariem.boudaya@yahoo.fr

**Keywords:** smartphone, children, sleep, nocturnal screen time, performance

## Abstract

Nocturnal smartphone use emits blue light, which can adversely affect sleep, leading to a variety of negative effects, particularly in children. Therefore, the present study aimed to determine the effect of acute (AC) (one night) and repeated (RC) (five nights) nocturnal smartphone exposure on sleep, cortisol, and next-day performance in Tunisian children. Thirteen participants (seven girls and six boys, age 9 ± 0.6, height 1.32 ± 0.06, weight 34.47 ± 4.41) attended six experimental nights. The experiment started with a baseline night (BL) with no smartphone exposure, followed by repeated sessions of nocturnal smartphone exposure lasting 90 minutes (08:00 pm–09:30 pm). Actigraphy; salivary cortisol; the Stroop test (selective attention); choice reaction time (CRT); N-back (working memory); counter-movement jump (CMJ), composed of flight time (time spent in the CMJ flight phase) and jump height; and a 30 m sprint were assessed the morning after each condition. Both AC and RC shortened total sleep time (TST) (*p* < 0.01), with a greater decrease with RC (−46.7 min, ∆% = −9.46) than AC (−28.8 min, ∆% = −5.8) compared to BL. AC and RC significantly increased waking after sleep onset (3.5 min, ∆% = 15.05, to 9.9 min, ∆% = 43.11%) and number of errors made on the Stroop test (1.8 error, ∆% = 74.23, to 3.07 error, ∆% = 97.56%). Children made 0.15 and 0.8 more errors (∆% = 6.2 to 57.61%) and spent 46.9 s and 71.6 s more time on CRT tasks (∆% = 7.22 to 11.11%) with AC and RC, respectively, compared to BL. The high-interference index of the Stroop task, CMJ performance, and 30 m sprint speed were only altered (*p* < 0.01) following RC (0.36, Δ% = 41.52%; −34 s, Δ% = −9.29%, for flight time and −1.23 m, −8.72%, for jump height; 0.49 s, Δ% = 6.48, respectively) when compared to BL. In conclusion, one- or five-night exposure to smartphones disturbed the children’s sleep quality and their performance, with more pronounced effects following RC.

## 1. Introduction

Circadian rhythms are biological cycles that follow a daily cycle. Cognitive and physical performance, sleep duration and quality, hormonal levels, and other physiological variables oscillate over a 24 h period. They are mostly influenced by the amount of light and darkness in an organism’s surroundings [1]. Any type of sleep disturbance can have an impact on an individual’s circadian rhythms, including children’s [2].

Previous chronobiological studies emphasized the importance of light stimulation for the synchronization of circadian rhythms in humans, particularly sleep rhythms. They found that using various electronic devices before bedtime adversely affects physiology, behavior, sleep, and circadian rhythms [3]. Indeed, the effect of artificial light at night (ALAN) on circadian rhythms depends on light intensity [4], duration and time of exposure [5], and the wavelength of the light [6].

Currently, light-emitting diodes (LEDs) are used with the majority of screens in digital media devices, with modern technology emitting mostly high-intensity and short-wavelength illumination [7]. At the level of the retina, intrinsically photosensitive ganglion cells (ipRGCs, expressing the photopigment melanopsin) are responsible for non-visual responses, such as the synchronization of the central circadian clock, pupil constriction, sleep architecture, and melatonin suppression [8,9]. These retinal photoreceptors transmit light information to the hypothalamus, specifically the suprachiasmatic nucleus, which is known as the master circadian clock and regulates hormone secretion, physiological rhythms, behavior, sleep, metabolism, immunity, alertness, and performance [10,11]. These photoreceptors are the most sensitive to blue monochromatic light (~480 nm) emitted by different digital devices [12]. Therefore, the huge rise in the use of different electronic devices across all ages can affect sleep [13], essentially when used in the evening [14]. A previous study demonstrated that illumination at only ~100 lux affects melatonin and cortisol secretion and disturbs circadian phases [15]. Furthermore, children exposed to dim light intensities (5–10 lux) before bedtime experience 82% melatonin suppression, which leads to the development and maintenance of behavioral sleep problems through impacts on the circadian timing system. [16]. These effects, particularly prominent in children, are primarily attributed to the heightened transmittance of their crystalline lens, especially due to short-wavelength light exposure [17].

Nowadays, electronic devices have become daily essentials, even for children [18]. One of the current major concerns is the impact of excessive use of and frequent consumption of content on digital devices on children’s well-being. Previous reviews of the literature found that media use affects children’s mental health [19], sleep quantity and quality [20], and physical health [21]. Blue light from screens can disrupt sleep hygiene, resulting in fatigue, lack of energy, and difficulties in concentration [22]. This harmful light not only damages children’s eyes but also disturbs their circadian cycles and inhibits melatonin secretion [23]. Additionally, children are more sensitive to light at night than adults [16]. Higuchi et al. [24] reported that school-aged children showed almost twice as much melatonin suppression during evening bright-light exposure (580 lux).

Apart from the effects of blue light, it is crucial to consider screen content and the manner in which children engage with screens [25]. The mechanisms underlying adverse health outcomes related to screen time and the specific contributions of different types of screen and media content to these outcomes remain unclear [26]. A rare study in this field specifically addressed the nature of the content viewed and indicated that reducing exposure to violent media content and not restricting screen time can improve children’s behavior [27]. Notably, video games and violent content consistently correlate with increased somatic complaints, aggressive behavior, and reduced sleep duration [28]. Although the use of all forms of electronic devices is associated with sleep difficulties, it is noteworthy that only the more active uses, such as social messaging and playing video games, are linked to shorter sleep durations [29].

In this regard, previous research has found that any disruption in sleep has a negative impact on both physical [30] and cognitive [31] performance. Indeed, sleep deprivation degrades waking neurobehavioral functions such as attention, cognitive ability, and memory [32]. In addition, most previous studies that employed sleep deprivation or restriction protocols found that reducing sleep time negatively affected athletic performance [33].

Tunisian children, like children all over the world, spend a lot of time in front of electronic devices. They spend an average of 1.53 h per day interacting with various displays, which serves as their primary source of entertainment [34]. However, there is currently a lack of information linking nocturnal screen time to children’s sleep quality and next-day performance [20]. Therefore, the present study aimed to inquire into the effect of acute (one night) and chronic (five nights) nocturnal smartphone exposure on cortisol, sleep quality, and next-day cognitive and physical performance.

We hypothesized that (i) a single session of nocturnal smartphone use would impact sleep and next-day performance and (ii) five consecutive sessions of nocturnal exposure to a smartphone could be more deleterious than acute exposure.

## 2. Materials and Methods

### 2.1. Participants

Thirteen healthy children (seven girls and six boys) (age 9 ± 0.6 (range 8 to 10 years); height 1.32 ± 0.06; weight 34.47 ± 4.41) from an urban school participated in this study. Participants and their parents were first extensively informed about the study procedure and materials. Parents provided informed consent, and children expressed their assent to participate. Potential study participants filled out questionnaires about their general health, sleep quality (Pittsburgh sleep quality index (PSQI)), and chronotype [35]. During the month preceding the experiment, a sleep diary was maintained (retiring and rising time, time in bed, sleep latency, waking frequency and duration, etc.). Only participants with good sleep quality (PSQI score < 5), and no extreme chronotype were recruited (PSQI score 2.15 ± 1.28; morningness–eveningness score 16.61 ± 0.86, distinctness of rhythm 15.15 ± 1.14). All subjects were prepubescent [36] with no history of medical or psychiatric treatments. Medication, caffeinated and soft drinks, and dark chocolate were not allowed for at least one week before and during the investigation. All children were not excessive screen consumers (daily screen time 1.22 h ± 0.22 h). Screen time was assessed by a parent’s report, in which they determined the total number of hours that the child spent in front of a computer, tablet, laptop, console, or TV. If screen time exceeded two hours per day, its consumption was considered excessive [37]. The study was conducted in accordance with the Declaration of Helsinki for human experimentation and was approved by the local Institutional Review Board (CPP SUD N° 0578/20).

### 2.2. Experimental Design

Children were recruited from a Tunisian primary school. Recruitment within the school was conducted through a structured process commencing with face-to-face meetings with parents and children after obtaining approval from school administrators. We conducted interviews with the parents to rule out major exclusion criteria (age, general health, daily screen time). The age for inclusion was set between 8 and 12 years old. Then, signed consent forms were collected from volunteering parents who were willing to allow their children to participate. None of the children in our study wore corrective lenses or glasses. One month before the experiment, participants completed a survey about chronotype and received a sleep diary to assess their sleep patterns (quantity, quality) and decide about their enrollment in the experiment. Any nocturnal screen time was prohibited one week prior to the experiment. One week before and throughout the protocol, each subject kept note of their standard times for eating (breakfast at 07:00 h, lunch at 12:00 h and dinner at 19:30 h) and sleeping habits (sleeping between 21:30 and 06:30 h) aligning with the typical daily routines of the subjects. The protocol was carried out during school days. Throughout the protocol, parents and educators were instructed to avoid subjecting children to stressful circumstances, conflicts, or intense emotions that could influence the outcomes.

The study began with a reference night considered a baseline night (BL) with no nocturnal screen time. The children were then subjected to five consecutive experimental nights (nights 2–6) of nocturnal smartphone exposure. The second night was considered the acute ALAN condition (AC) and the sixth night was considered the repeated ALAN condition (RC). All light exposure sessions were administered at the same time and each one lasted for ninety minutes (from 8 pm to 9.30 pm). One of the two parents provided the smartphone to their child. The experimenters were responsible for visiting the children’s homes during the protocol to oversee task administration, ensure the same smartphone and room were used each night, and maintain a consistent distance between the smartphone and the subjects before each exposure. The nocturnal exposure to the smartphone was conducted in the child’s home instead of the lab to mitigate potential stress or anxiety associated with unfamiliar environments and to minimize external influences and distractions, especially considering the timing of the exposure close to bedtime.

During the study, participants wore an accelerometer (Actilife, Actigraph), and morning measurements (cortisol, physical and cognitive performance) were taken following BL, AC, and RC nights. The administration of cognitive and physical performance tasks was carried out by experimenters at the school who were actively engaged in the study and well-informed about the materials and tasks used. Performance assessment was consistently conducted at the same time of day (between 11:00 am and 11:30 am) and followed the same order for each session, with cognitive tests preceding the physical tests. A 5 min rest interval was provided between the cognitive and physical tests. On each exposure night, the children attended an online classmate meeting. The initiation of the online meeting was conducted by a teacher assigned this responsibility to ensure consistency, proper timing, and alignment with the relevant content.

Considering the variety of effects influenced by various potential mechanisms, including different time displacement, psychological stimulation from content, and the alerting and circadian effects of light, it was essential to control these factors. This acknowledgment validates our choice to standardize content for children as non-aggressive and mentally non-fatiguing. The screen time exposure consisted of an interactive and engaging online meeting during which children could interact with their classmates through various activities, games, and/or discussions under the guidance of the teacher who ensured the participation of all participants.

Throughout the exposure, volunteers were seated in front of the high-brightness smartphone (adjusted at 450 lux) with ambient light (fixed intensity of 500 lux) provided by an incandescent lamp (in order to eliminate any source of LED light) measured by an android m-app (Light Meter11 Trajkovski Labs app) that uses the light sensor [38]. The measurements were conducted at night to minimize light reflection and avoid the interference of other light sources. [39].

**Figure 1:**
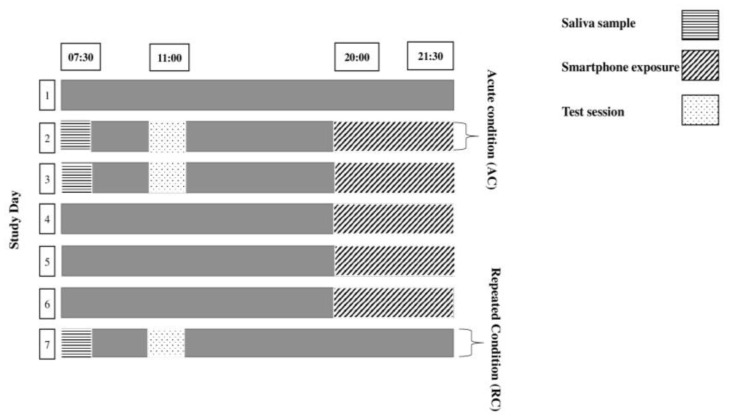
Experimental design.

During and one week prior to the experiment, participants were instructed to keep a regular sleep–wake schedule (bedtimes and wake times within 30 min of the self-selected target time). Additionally, children were not allowed to nap or exercise and did not have access to any digital devices during the daytime (Figure 1). The verification of their adherence to these guidelines was achieved through parental and teachers’ reports and daily logs.

### 2.3. Measurements

#### 2.3.1. Actigraphy

For objective sleep measurements, participants wore an ActiGraphy GT3X-BT (ActiGraph Corporation, Pensacola, FL, USA) on their non-dominant arm. It was used as the main measure of sleep timing, duration, and quality. Long established as a valid and reliable sleep assessment method, actigraphy is able to objectively evaluate sleep–wake patterns in the child’s home environment [40]. The actigraphy score permits the identification of periods of sleep and wakefulness [41]. Actigraphs continuously recorded the movements of children during the night, and these data were later translated into sleep metrics using Sadeh’s validated algorithm for children [42].

To deduce the daily phases of sleep and activity, these records were translated to different scores for total sleep time (TST) from sleep onset to final waking; time in bed (TIB) from lights out to getting out of bed events; sleep efficiency (SE), the ratio of total sleep duration to the total time spent in bed; sleep onset latency (SOL), minutes from bedtime to the initial 20 min sleep period; and wake after sleep onset (WASO), the amount of time spent awake between sleep onset and offset [43]. Parents were instructed to attach these wristwatch-like devices to the child’s nondominant wrist during the protocol. In addition, parents completed a sleep diary recording their child’s bedtime and getting-up time during the study. These diary parameters were used to support calculations and analyses of actigraphy data.

#### 2.3.2. Cortisol Measurements

In the current study, levels of cortisol were measured in saliva. It has been established that salivary levels closely match those found in corresponding blood samples [44]. Saliva samples were collected 30 min after the child was awakened while keeping the child fasting [45]. Taking saliva for this analysis required several precautions to avoid any interference that could distort the test results. Parents were informed that their children should not brush their teeth prior to collection. Children had to spit directly into the dry test tubes labelled with the relevant information. The samples were kept at ambient temperature and immediately sent to the biochemistry laboratory of Hedi Chaker University Hospital in Sfax.

The collected saliva samples were centrifuged for 15 min at 3000 revolutions/min, and then the supernatant was taken from the Eppendorf tubes and frozen at −20 °C. On the day of the analysis, the samples were thawed to room temperature (20 to 25 °C) and then vortexed and analyzed. Salivary cortisol was assessed using electrochemiluminescence assays (ECLIA) with the COBAS 6000 automated system produced by Roche Diagnostics (Tokyo, Japan), and all relevant diagnostic reagents from the same company were used. The unit of measurement for cortisol is nanograms per milliliter (ng/mL).

#### 2.3.3. Cognitive Measures

##### Stroop Task

To evaluate selective attention, participants underwent the paper-and-pencil Victoria version of the Stroop test, comprising three cards. Each card contained six rows of four items [46,47]. Four colors were used: blue, green, yellow, and red. For card 1 (M), children were asked to verbally name a series of color words written in different colors (word task). This component is believed to reflect the basic reading rate and may be affected by speech, motor problems, or learning disabilities. For card 2 (C), participants were required to identify and name the color of a bar (color task). Card 3 (I) featured the color–word task, where individuals were presented with color names printed in conflicting ink colors (e.g., the word “blue” in red ink) and instructed to name the color of the ink rather than the word. Children were required to respond as rapidly as possible. The examiner timed the naming speed for all cards. The low interference index refers to the performance under a congruent condition (time card M/time card C) and the high interference index refers to the performance under an incongruent condition (time card I/time card C), and the number of errors made (“color errors” and ”interfering errors”) was analyzed. Interference scores reflect the extent to which participants are able to shift perceptual sets to conform to changing demands [48].

##### Choice Reaction Time

The choice reaction time (CRT) test (using F. Talkin’s test) was performed using Open Sesame software (version 3.1) [49] to assess the speed of cognitive processing and reaction time. Pairs of colored geometric shapes were initially presented to the subject and considered as “targets”. For each form, the correct key on the computer had to be pressed when the right form appeared, and the computer calculated the RT. Each subject had 10 target presentations. Then, the program displayed the average score along with the number of errors. Scores are expressed in milliseconds (msec), with higher times reflecting poorer performance [50].

##### N-Back Task

In this study, we use the n-back task programmed by Robinson and Fuller [51] via computer [52] to assess working memory and attention. The task version that was used was 1-back. We used words as stimuli in all blocks composed of 20 consonants. They were presented one by one in the center of the screen. When the subsequent letter was the same as that from two trials ago, they had to press “yes”; otherwise, they had to press the “no” key. The children had 3.5 s, from stimulus onset until the beginning of the subsequent trial, to press the corresponding response key. For each key press, it was determined if it was a correct response or an incorrect response (number of errors).

#### 2.3.4. Physical Measures

##### Counter-Movement Jump

Counter-movement jump (CMJ) is a vertical jump often used as a field or laboratory test to study the stretching–relaxing cycle of the lower limbs. The starting position is standing with hands placed on the hips, allowing the subjects to jump higher. Children performed a bending of the lower limbs (this is the counter-movement) immediately followed by a complete extension of the lower limbs. During this jump, the subject was instructed to jump as high as possible. The height of the jump was measured using an Optojump system (Optojump, Microgate SRL, Bolzano, Italy). The Optojump system measures the flight time with an accuracy of 1/1000 s (1 kHz), which refers to the duration for which the subject is in the air while performing the vertical jumps. Three CMJs were performed with a 1 min interval between each, and the trial yielding the best performance was selected for subsequent statistical analysis. The jump height was estimated as a 9.81 flight time times 2/8 [53].

##### Sprint Test

The 30 m sprint is often performed by children and adolescents [54]. It consists of covering as quickly as possible a distance of 30 m in a corridor. This distance is related to a stopwatch for calculating speed [55]. Three trials were completed, and the best-performing trial was used for the subsequent statistical analysis.

### 2.4. Statistical Analysis

All data are presented as means and standard deviations (±SD). Statistical analysis was performed using SPSS statistical software (v.23, IBM, New York, NY, USA) and figures were created using GraphPad Prism 8 (GraphPad Software, San Diego, CA, USA). The normality assumption was verified using the Shapiro–Wilk test. One-way repeated-measures ANOVA was used to assess the main effect of the condition and two-tailed t-tests were used to compare pairwise conditions (BL vs. AC, BL vs. RC, and AC vs. RC). If significant main effects were detected, a Bonferroni post hoc test was used to compare pairwise conditions (BL vs. AC, BL vs. RC, and AC vs. RC). Effect sizes were calculated through partial eta-squared (ηp^2^) for the one-way repeated-measures ANOVA and interpreted according to Cohen’s classification (i.e., trivial effect sizes <0.2, small effect sizes <0.5, moderate effect sizes <0.8, and large effect sizes ≥0.8). Statistical significance level was set at *p* < 0.05.

## 3. Results

### 3.1. Objective Sleep Measurements

The results for sleep patterns for BL, AC, and RC are summarized in Table 1. ANOVA showed a significant main effect of nocturnal smartphone exposures on TST (F(2,26) = 11.16; *p* = 0.01; ηp^2^ = 0.4) and WASO (F(2,26) = 19.02; *p* < 0.001; ηp^2^ = 0.83). No significant effect of nocturnal smartphone exposure was observed on SOL, SE, or TIB. The post hoc test revealed a greater decrease in TST values for RC (∆% = −9.46) compared to BL (*p* < 0.01) and a greater increase in WASO values for AC and RC (∆% = 15.05 and 43.11, respectively) compared to BL (*p* = 0.013 and *p* < 0.001, respectively). Interestingly, WASO also increased for RC compared to AC (*p* < 0.01).

### 3.2. Salivary Cortisol

As Table 2 shows, there were no significant changes in salivary cortisol 30 min after awakening following AC and RC exposure (*p* > 0.05).

### 3.3. Cognitive Performance

**Figure 2:**
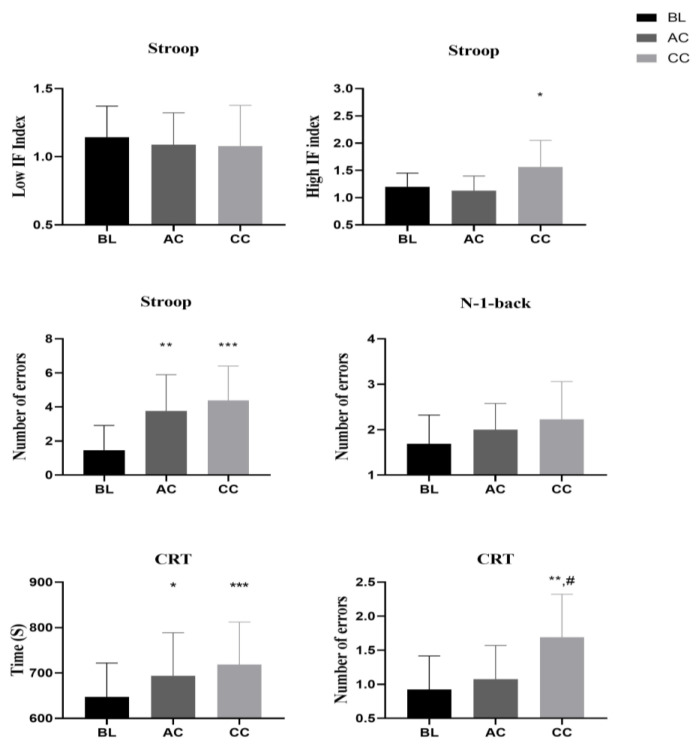
Cognitive performance after BL, AC, and RC. BL: baseline night, AC: acute condition, RC: repeated condition, CRT: choice reaction time. *, **, ***: significant difference from baseline (*p* < 0.05, *p* < 0.01, and *p* < 0.001, respectively). #: significant difference between AC and RC nights (*p* < 0.05).

Data related to cognitive performance are presented in Figure 2. The ANOVA showed significant main effects of nocturnal smartphone exposures on time (F(2,26) = 13; *p* < 0.001; ηp^2^ = 0.52) and number of errors made on the CRT test (F(2,26) = 7.35; *p* < 0.01; ηp^2^ = 0.38). There was a significant effect on number of errors made on the Stroop test after the AC and RC (*p* < 0.01, *p* < 0.001, ∆% = 74.23%, 97.56%, respectively) and a significant effect of the RC on the high interference index (*p* < 0.05, ∆% =+ 41.52%). The post hoc test revealed a significant increase in time mode on the CRT test after the AC (∆% =+ 7.22%) and RC (∆% =+ 11.11%) compared to BL (*p* < 0.03 and *p* < 0.001, respectively) and a significant increase in the number of errors on the CRT test for the RC and AC (∆% =+ 57.62; 6.2%) compared to BL (*p* = 0.018 and *p* = 0.041, respectively). However, there was no significant effect from the AC and RC in the N-1-back task (*p* > 0.05).

### 3.4. Physical Performance

The summary statistics related to physical performance are presented in Table 3. Statistical analyses (ANOVA) demonstrated a significant effect of exposure on CMJ flight time (F(2,26) = 6.44; *p* < 0.01; ηp^2^ = 0.35), jump height (F(2,26) = 5.24; *p* = 0.013; ηp^2^ = 0.3), and 30 m speed (F(2,26) = 5.27; *p* = 0.013; ηp^2^= 0.3). The post hoc test showed a greater decrease in jump height for the RC compared to BL (*p* = 0.015, ∆% = −8.72%). CMJ flight time and 30 m speed decreased significantly for the RC (∆% = −9.29 and 6.48 respectively) compared to BL (*p* < 0.05).

## 4. Discussion

This study sought to determine the effect of nocturnal smartphone exposure on sleep, salivary cortisol, and next-day performance in children. The main findings indicate that TST and WASO patterns were negatively affected by nocturnal smartphone exposure. These findings are consistent with many previous studies that have shown that nocturnal smartphone use is associated with restless sleep, difficulty falling asleep, and fatigue [22]. This exposure has also been linked to shorter sleep duration and worse sleep quality due to the suppression of melatonin [56]. In line with our study, existing data show that both acute and repeated nocturnal use of computer screens disrupts the continuity and architecture of sleep as measured with polysomnographic measurements in adults [57]. In addition, one minute of smartphone usage was shown to be associated with an increase of 0.32 min in WASO [58]. Importantly, following nocturnal exposure to different light intensities (between 5 and 5000 lux), children showed significant circadian phase delays associated with a greater percentage of melatonin suppression, which can contribute to behavioral sleep difficulties [59].

While the existing body of literature has extensively explored the role of light emitted from screens in influencing sleep patterns, there is a growing body of evidence suggesting that the content displayed on screens may contribute to varying degrees of sleep disturbances [60]. However, the impact of screen content on sleep outcomes is nuanced, with mixed results regarding factors such as type, time displacement, interactivity level, and psychological stimulation [61]. Studies diverge on whether interactive screen media use, including activities like video games and mobile devices, has a more significant impact on sleep than passive use [62]. Additionally, research findings indicate that video games, particularly those of a violent nature, are associated with increased heart rate, slightly delayed sleep onset, and decreased rapid eye movement during sleep [63]. Consistent with our findings, sleep-related issues persist even in instances of non-violent media use [64]. These diverse findings highlight the complexity of the relationship between screen content and its influence on sleep outcomes. They highlight the necessity for further investigation to comprehend the multitude of factors involved.

The present study showed that concentrations of cortisol 30 min after awakening were not affected by either acute or repeated nocturnal smartphone use. Most of the previous studies have shown contradictory results regarding the effect of light on cortisol secretion. Indeed, they have demonstrated that cortisol levels may increase [65], decrease [66], or remain unchanged [67] due to light increase. This discrepancy may be due to the differences in light intensity, duration, and time of exposure. As such, future studies could establish the effect of different types of ALAN on awakening cortisol levels.

Our findings show that nocturnal exposure to ALAN, whether for just one night or five nights, affects selective attention and reaction time. These effects are especially concerning in children because attention ability and reaction time are critical cognitive aspects used in the majority of daily tasks, such as sports and academic work [68]. In general, the increasing use of all types of digital devices is correlated with negative effects on children’s sleep duration, academic achievements, and cognitive development [69].

Since nighttime exposure to blue light is a sleep disruptor [70], this disturbance can have many effects on cognitive performance. A previous study demonstrated that sleep disturbances degrade waking neurobehavioral functions such as drowsiness, attention, cognitive ability, and memory [32].

The current findings showed that one-time nocturnal smartphone exposure had no effect on any physical performance measures. However, repeated nocturnal exposure altered jumping and sprint performance. Studies that focus on ALAN effects on physical performance remain very limited, with most of the previous research focusing more on the relationship between screen time, sedentary lifestyles, and obesity [71]. Indeed, daily physical activity is negatively associated with screen time in children [72]. Children who spent more time in front of the TV showed lower physical performance in terms of leg extension, strength, and pull-ups [73]. In line with the present findings, a recent study showed that acute exposure to an electronic device (tablet) before bedtime affected sleepiness and melatonin concentration but not the next day’s performance in athletes [74].

The present deterioration in physical performance following the five-night smartphone use can be linked to the deeper alteration of TST and WASO following the fifth night. In this context, it is well established that an alteration in sleep quality and/or quantity can affect physical performance the next day. Indeed, reduced sleep time has been shown to alter aerobic capacity [75], sub-maximum force [76], perceived effort [77], and reaction time [74]. While our study allowed for within-subject and pre–post comparison, the lack of a control group and the randomization of the condition order constrain the generalizability of our findings. Future research should take these limitations into consideration.

Interestingly, the present findings showed that increasing the number of exposure nights from one to five deepened the negative effect of smartphone exposure on some sleep and cognitive parameters (e.g., WASO and number of errors in CRT) and most of the measured physical performance (i.e., CMJ flight time and 30 m speed). Therefore, to maintain children’s physical performance, repeated nocturnal smartphone exposure must be avoided, particularly during holidays and weekends, as these periods are associated with increased sedentary behavior and screen time [78,79]. Additionally, these findings suggest that an increased number of exposure nights amplifies the effects of screen exposure on children’s sleep and physical and cognitive performance and can thereby explain previous inconsistencies between findings.

## 5. Strengths, Limitations, and Perspectives

To the best of our knowledge, this is the first study to examine the acute and repeated effects of nocturnal smartphone use on sleep and next-day performance in children. Comparing the influence of non-exposure to acute and repeated exposure to nocturnal illumination emerging from smartphones on sleep and daily functions in children remains necessary to evaluate objectively the different effects of nocturnal screen time.

However, this study has some limitations that should be considered when interpreting the findings. Indeed, the number of subjects was quite small, only one duration and one type of exposure were tested, and the measurements of light intensity obtained with the smartphone application were not compared with values obtained with real lux meter indicator equipment. Therefore, further large-scale comparative studies investigating different durations and types of exposure to digital devices are needed to provide more detailed information about the impact of self-luminous displays. In these studies, it is advised to use a real lux meter indicator as a guiding reference for luminance levels [80]. A significant limitation of our study was the lack of a control group and the non-randomization of the condition order, which may have led to order effects. In addition, future research should aim for more comprehensive and thorough examination of the diverse variables, including school demands, social interactions, family dynamics, anxiety, and stress, that may influence the outcomes.

## 6. Conclusions

In conclusion, this study suggests that one- or five-night exposure to the blue light display of smartphones may disrupt children’s sleep quality and their cognitive performance, with more pronounced effects following the five-night protocol. Children’s physical performance was only altered following the five-night protocol. To maintain children’s sleep quality and physical and cognitive performance, nocturnal smartphone exposure, particularly repeated exposure over the weekend or holidays, must be avoided.

## Figures and Tables

**Table 1:** Sleep patterns for the BL night and after AC and RC exposure.

	BL	AC	RC	ANOVA (F(2,26), *p*, ηp^2^)
**SOL (min)**	12.62 ± 9.82	14.15 ± 6.97	12.77 ± 15.27	(0.072, 0.93, 0.21)
**SE (%)**	88.14 ± 5.66	85.59 ± 7.17	81.5 ± 2.12	(2.87, 0.076, 0.22)
**TIB (min)**	551.23 ± 26.18	541.46 ± 22.53	541.46 ± 24.28	(0.71, 0.49, 0.04)
**TST (min)**	487.08 ± 40.2	458.38 ± 40.9 ***	440.08 ± 38.92 ***	(11.16, 0.01, 0.4)
**WASO (min)**	23.69 ± 2.21	27.23 ± 4.23 **	33.62 ± 5.38 ***^,##^	(19.02, <0.001, 0.83)

BL: baseline night, AC: acute condition, RC: repeated condition, SOL: sleep onset latency, SE: sleep efficiency, TIB: time in bed, TST: total sleep time, WASO: wake after sleep onset. **, ***: significant difference from baseline (*p* < 0.01 and *p* < 0.001, respectively). ^##^: significant difference between AC and RC (*p* < 0.01).

**Table 2:** Salivary cortisol concentrations after BL night and after AC and RC exposure.

	BL	AC	RC	ANOVA (F(1,13), *p*, ηp^2^)
**Cortisol** **(ng/mL)**	3.48 ± 1.97	3.42 ± 1.69	2.69 ± 0.89	(1.96, *p* = 0.19, 0.26)

BL: baseline night, AC: acute condition, RC: repeated condition.

**Table 3:** Physical performance for BL night and after AC and RC exposure.

	BL	AC	RC	ANOVA (F(2,26), *p*, ηp^2^)
**CMJ**				
**Flight time (s)**	0.36 ± 0.04	0.36 ± 0.01	0.32 ± 0.04 *^,#^	(6.44, *p* < 0.01, 0.35)
**Jump height (cm)**	14.08 ± 1.2	13.79 ± 1.55	12.85 ± 1.63 **	(5.24, 0.013, 0.3)
**30 m sprint speed**				
**Time (s)**	7.31 ± 0.56	7.38 ± 0.49	7.75 ± 0.34 *^,#^	(5.27, 0.013, 0.3)

BL: baseline night, AC: acute condition, RC: repeated condition, CMJ: counter-movement jump. *, **: significant difference from baseline (*p* < 0.05 and *p* < 0.01, respectively). ^#^: significant difference between AC and RC (*p* < 0.05).

## Data Availability

Data are available from the first author upon reasonable request.
